# The role of glycolytic metabolic pathways in cardiovascular disease and potential therapeutic approaches

**DOI:** 10.1007/s00395-023-01018-w

**Published:** 2023-11-08

**Authors:** Shuxian Chen, Yuanming Zou, Chunyu Song, Kexin Cao, Kexin Cai, Yanjiao Wu, Zhaobo Zhang, Danxi Geng, Wei Sun, Nanxiang Ouyang, Naijin Zhang, Zhao Li, Guozhe Sun, Yixiao Zhang, Yingxian Sun, Ying Zhang

**Affiliations:** 1 Department of Cardiology, The First Hospital of China Medical University, 155 Nanjing North Street, Heping District, Shenyang, 110001 Liaoning Province People’s Republic of China; 2 Department of Thyroid Surgery, The First Hospital of China Medical University, 155 Nanjing North Street, Heping District, Shenyang, 110001 Liaoning Province People’s Republic of China; 3 Institute of Health Sciences, China Medical University, 77 Puhe Road, Shenbei New District, Shenyang, 110122 Liaoning Province People’s Republic of China; 4 Key Laboratory of Reproductive and Genetic Medicine, China Medical University, National Health Commission, 77 Puhe Road, Shenbei New District, Shenyang, 110122 Liaoning Province People’s Republic of China; 5 Department of Urology Surgery, Shengjing Hospital of China Medical University, 36 Sanhao Street, Heping District, Shenyang, 110004 Liaoning Province People’s Republic of China

**Keywords:** Glycolysis, Metabolism, Heart failure, Ischemia–reperfusion, Cardiovascular disease

## Abstract

Cardiovascular disease (CVD) is a major threat to human health, accounting for 46% of non-communicable disease deaths. Glycolysis is a conserved and rigorous biological process that breaks down glucose into pyruvate, and its primary function is to provide the body with the energy and intermediate products needed for life activities. The non-glycolytic actions of enzymes associated with the glycolytic pathway have long been found to be associated with the development of CVD, typically exemplified by metabolic remodeling in heart failure, which is a condition in which the heart exhibits a rapid adaptive response to hypoxic and hypoxic conditions, occurring early in the course of heart failure. It is mainly characterized by a decrease in oxidative phosphorylation and a rise in the glycolytic pathway, and the rise in glycolysis is considered a hallmark of metabolic remodeling. In addition to this, the glycolytic metabolic pathway is the main source of energy for cardiomyocytes during ischemia–reperfusion. Not only that, the auxiliary pathways of glycolysis, such as the polyol pathway, hexosamine pathway, and pentose phosphate pathway, are also closely related to CVD. Therefore, targeting glycolysis is very attractive for therapeutic intervention in CVD. However, the relationship between glycolytic pathway and CVD is very complex, and some preclinical studies have confirmed that targeting glycolysis does have a certain degree of efficacy, but its specific role in the development of CVD has yet to be explored. This article aims to summarize the current knowledge regarding the glycolytic pathway and its key enzymes (including hexokinase (HK), phosphoglucose isomerase (PGI), phosphofructokinase-1 (PFK1), aldolase (Aldolase), phosphoglycerate metatase (PGAM), enolase (ENO) pyruvate kinase (PKM) lactate dehydrogenase (LDH)) for their role in cardiovascular diseases (e.g., heart failure, myocardial infarction, atherosclerosis) and possible emerging therapeutic targets.

## Introduction

Cardiovascular disease (CVD) is one of the major causes of the global disease burden, and it is important to develop effective and timely strategies to address the challenges of the CVD epidemic. Risk factors associated with CVD include obesity, lack of physical activity, poor diet, diabetes, smoking, etc. [1, 2]. Therapeutic strategies for CVD are categorized into approaches that lower LDL, lower triglycerides, lower Lp(a), and elevated HDL [3]. These involve drugs such as antiplatelet agents, statins, beta-blockers, and angiotensin-converting enzyme inhibitors, the use of which has become the cornerstone of treatment for CVD. significant reductions in CVD events and mortality have been observed [3, 4]. These drugs can significantly reduce the risk of adverse cardiovascular events in patients [5], which is important for improving patient prognosis. However, with the progressive increase in the prevalence and mortality of CVD, an urgent search for therapeutic means to address the disease beyond conventional approaches is important to alleviate medical stress. In recent years, glycolytic dysregulation has been noted in many pathological processes of CVD, and the exploration of glycolytic pathways has provided many new potential therapeutic directions for the treatment of CVD. Nevertheless, the relevant molecules as well as pathways involved in the role of glycolytic reprogramming are not well understood [6]. Therefore, continued exploration of the glycolytic pathway is necessary.

Glycolysis, which occurs in the cytoplasm, is the process of breaking down glucose to produce pyruvate, and it is the most critical pathway of glucose metabolism in the body [7], involving the linkage of energy metabolism, homeostasis, and various physiological functions, such as muscle movement, development, and neurotransmission [8]. It is also capable of producing pyruvate and limited adenosine triphosphate (ATP) to meet the energy demands. Glycolytic pathway is achieved through the oxygen non-dependent activity of ten metabolic enzymes and involves three rate-limiting enzymes, including hexokinase 2 (HK2), phosphofructokinase 1 (PFK1) and pyruvate kinase type M2 (PKM2) [9]. The process can be divided into three phases, which are the initiation phase (1 molecule of glucose to 1 molecule of F-1,6-2P), the cleavage phase (1 molecule of F-1,6-2P cleaved to 1 molecule of DHAP(Dihydroxyacetone phosphate) and 1 molecule of PGAL(3-phosphoglyceraldehyde) and the redox phase (2 molecules of PGAL reduced to 2 molecules of pyruvate). Under aerobic conditions, pyruvate enters the mitochondria and participates in the tricarboxylic acid cycle, where it is oxidized to CO2 and H2O. When mitochondrial oxidative respiration is impaired, pyruvate is then reduced to lactate by NADH, which is subsequently taken up by most tissues of the body for recycling and metabolism to pyruvate and downstream TCA intermediates [10].

Furthermore, glycolysis not only serves as a source of energy for cellular metabolism but also generates metabolites that play a crucial role in cellular function [11]. In cardiomyocytes, glucose is first converted to glucose-6-phosphate (G6P) by hexokinase, and can subsequently undergo various metabolic pathways, such as glycolysis, pentose phosphate pathway (PPP), hexosamine synthesis pathway (HBP), and serine biosynthesis pathway [12]. Excessive glucose flux through these pathways is associated with oxidative stress as well as the development of cardiovascular complications [13].

In HF, the compensatory response to the decrease in mitochondrial oxidative metabolism and ATP production is a rise in the glycolytic response. In this process, the expression of the glycolytic intermediate GLUT1 glucose transporter protein increases, as does the activity of PFK-1 and glycolytic flux. However, the slightly elevated energy produced by glycolysis was not able to completely rescue the cardiac dysfunction caused by energy failure, but instead was able to exacerbate HF by activating metabolic pathways branching off from glycolysis (e.g., polyol and hexosamine biosynthesis pathways) leading to myocardial remodeling [14, 15]. Therefore, it is important to assess glycolysis from the perspective of glycolytic intermediates as well as glycolytic branching pathways (rather than the overall glycolysis) perspective it may be more valuable to assess the impact of altered glycolytic pathway activity on CVD.

## Glycolysis-related enzymes and cardiovascular disease

### Hexokinase

Hexokinases (HKs) catalyze the first step in glucose metabolism, phosphorylating glucose to glucose-6-phosphate (G6P). HKs are composed of four families of isoforms of which HK1 and HK2 are the most abundant.HK1 (“brain HK”) is ubiquitous in HK1 (“brain HK”) is ubiquitous in most tissues, especially the brain and erythrocytes. HK2 (“muscle HK”) is found mainly in insulin-sensitive tissues, such as adipocytes, adult skeletal muscle, and cardiac muscle [16]. The coordination of glucose’s catabolic and anabolic utilization is facilitated by HKs. Both HKI and HK2 contain hydrophobic amino-terminal mitochondrial-binding motifs, and HKI binds more strongly to mitochondria and promotes glucose metabolism. HK2, however, has a more complex role. When located in the cytoplasm, HK2 is able to direct G6P to the glycogen and pentose phosphate pathways in the cytoplasm, and when bound to mitochondria, it is preferentially used for glycolysis and oxidative phosphorylation [17]. HK3 and HK4 are present in the cytoplasm, lack mitochondrial binding motifs, and perform primarily anabolic functions. HK4 also can shuttle to the nucleus and may play a role in gene transcription/new protein synthesis [16]. HK1 is the predominant isoform in the embryonic, fetal, and neonatal heart and promotes glycolysis. After birth, glycolysis is diminished as the diet shifts from a glucose-sugar-based diet to a mixed carbohydrate-fat oral diet, conferring a preference for fatty acids, lactate, and ketone bodies in the adult heart. This preference switch has resulted in the predominance of insulin-regulated hexokinase II in the adult heart. insulin-regulated hexokinase II is predominant in the adult heart [18].

#### Myocardial infarction

The findings revealed that reduced HK2 levels were able to lead to increased cell death and increased myocardial fibrosis in mice after I/R injury [19]. Myocardial infarction (MI) is best treated by timely reperfusion, but reperfusion will further damage myocardial ischemia/reperfusion. An increase in glucose oxidation occurs during this process, leading to an abnormal accumulation of glycolytic intermediates that drive mitochondrial dysfunction and increased reactive oxygen species (ROS) formation [17, 20]. This condition known as metabolic overload, and the metabolic overload cell vulnerable to the damaging effects of hyperglycemia [21]. Hyperglycemia is a state of stress during acute myocardial infarction. Hyperglycemia increases cytoplasmic ROS concentrations by activating NADPH oxidase 2 and reduces HK2 binding to mitochondria by increasing cellular G6P levels [22]. During ischemia–reperfusion, as the metabolism of the myocardium shifts from oxidative phosphorylation to aerobic glycolysis, the G6P concentration increases three to tenfold [22]. The high concentration of G6P with acidosis is the trigger for HK2 dissociation from voltage-dependent anion channel (VDAC) [21, 23, 24]. This is an important cause of apoptosis and necrosis of cardiomyocytes in the ischemic heart. In parallel, reduced tissue oxygen and nutrients and increased ROS have also been found in the ischemic heart, all of which can further lead to apoptosis in cardiac myocytes [25]. The above results suggest that HK2 dissociates from cardiac mitochondria during ischemia and that the degree of its dissociation is an important influence on infarct size [26, 27].

It has been found that the N-terminal structural domain of HK2 binds to VDAC on the outer mitochondrial membrane (OMM), obtains ATP from the OMM, and phosphorylates glucose to G6P [23]. As mediators of the first enzymatic step of glucose metabolism, HKs are also able to coordinate various catabolic and anabolic uses of glucose and have a very important role in the treatment of myocardial infarction [24]. Mitochondrial permeability transition pore (mPTP) is a Ca^2+^-dependent channel formed between the inner and outer mitochondrial membranes. Sustained mPTP opening induces mitochondrial swelling and ATP depletion, ultimately leading to cell death [16, 28, 29]. When HK2 interacts directly with VDAC on OMM, it can prevent mPTP opening and thus exert anti-apoptotic effects [16, 30].

In the treatment of myocardial infarction, ischemic preconditioning slows the rate of ATP depletion during subsequent ischemic episodes and can benefit the myocardium by preventing large accumulation of metabolic breakdown products such as lactate and protons. This treatment is called Ischemic Preconditioning (IPC) [31]. It has been reported that IPC can phosphorylate HK2 at Thr-473 via the Akt- glycogen synthase kinase-3 (GSK3β) signaling pathway [23, 32], enhance the translocation of GLUT4 transporter protein to the cell membrane, and the translocation of HK2 from the cytoplasm to the mitochondria, and increase the binding of HK2 to the mitochondria [33, 34], thereby improving myocardial resistance to mPTP opening and cell death after reperfusion [16, 35]. The Akt-GSK3β pathway mentioned therein is an important pathway that regulates cardiomyocyte growth and survival. GSK3β can induce apoptosis by stimulating transcription factors, and GSK3β phosphorylation by Akt results in inhibition of mPTP opening, ultimately enhancing myocardial survival [36]. Hypothermia is widely used in coronary artery bypass grafting. Which activates the Akt signaling pathway by increasing the phosphorylation of Akt, promoting the binding of HK2 to mitochondria, and simultaneously decreasing cytochrome c release. In this way, it preserves mitochondrial and cardiac function, reduces cardiac energy demand, and exerts cardioprotective effects like those of IPC [37].

I/R is also able to induce VDAC1 oligomerization by activating power-related protein 1 (Drp1)-dependent mitochondrial fission to reduce VDAC1-HK2 interactions and promote mPTP opening, ultimately leading to mitochondrial autophagy-mediated cell death. This Drp1-dependent mitochondrial fission was found to be blunted by AMPKα, and melatonin was able to inhibit the mitochondrial fission-VDAC1-HK2- mPTP-mitochondrial autophagy axis and protect the cardiac microvascular system from IR by activating AMPKα [38]. Not only that, but some researchers also claim that mitochondrial exposure to peroxynitrite (ONOO-) leads to tyrosine nitration of adenine nucleotide transferase (ANT) and VDAC1 and induces VDAC1 oligomerization, leading to the separation of HK2 from VDAC1 and ultimately to increased mitochondrial dysfunction and severity of myocardial infarction [39]. MCGR (an antioxidant mixture) was found to be cardioprotective against I/R injury by preserving the binding of HK2 to mitochondria. In addition, a cell-permeable peptide TAT-HK2 (TAT peptide GRKKRRQRRRPQ, a trans-activator from HIV transcription) containing an HK2 mitochondrial binding motif was also found to induce HK2 translocation from mitochondria and exacerbate cardiac reperfusion injury [40, 41]. Overall, prevention of HK2 during ischemia–reperfusion dissociation from mitochondria could rescue cardiomyocyte apoptosis, which may be an important direction to reduce cardiac I/R injury in the future [35, 37].

Mitochondrial autophagy, a type of mitochondria-specific autophagy that eliminates damaged mitochondria, is an essential mechanism for controlling mitochondrial health in cardiomyocytes in both physiological and pathophysiological settings [42]. It has been claimed that moderate activation of autophagy during I/R has cardioprotective effects [43]. This activation requires the involvement of a multisubunit protein kinase complex, rapamycin complex 1 (mTORC1). Activation of mTORC1 promotes the biosynthesis of macromolecules (including proteins, lipids, and nucleic acids) while inhibiting catabolic processes [44]. Under nutrient-rich conditions, mTORC1 inhibits autophagy by phosphorylating unc-51-like kinase 1 (ULK1). mTORC1 activity is inhibited when cellular energy is low, while ULK1 is activated, thereby inducing autophagy [43, 45]. It has been shown that HK2 can inhibit mTORC1 activity during reperfusion by directly interacting with PPTOR, a component of mTORC1, through its TOS motif (mTOR signaling motif), promoting autophagy in response to glucose deprivation (HK substrate deprivation) to protect cardiomyocytes. However, this binding is inhibited by G6P generated by the catalytic activity of HK2 [45, 46]. Therefore, regulating the intracellular localization of HK2 and promoting its binding to mTORC1 may be a novel approach to regulate mitochondrial autophagy to prevent ischemic stress-induced cell death.

When glucose metabolism required to maintain cellular and tissue processes increases or decreases, it is regulated primarily through four key steps: glucose uptake, hexokinase, phosphofructokinase (PFK), and the import and export of lactate to meet metabolic needs, during which G6P(A glycolytic by-product) concentrations are tightly controlled and do not increase or decrease significantly. This process is known as scheduled glycolysis [21]. Recently, glycolytic overload has been found to exacerbate the progression of I/R in ischemia–reperfusion injury [47]. Ischemia–reperfusion injury is a disease associated with high concentrations of G6P. It is known that during ischemia, glycogen is rapidly broken down. And the concentration of G6P in the myocardium was significantly increased. This results in dissociation of HK2 from mitochondria and impaired ATP production, as well as mitochondrial membrane hyperpolarization, mitochondrial dysfunction, and increased formation of ROS, exacerbating the progression of I/R injury [21]. Naila Rabbani et al. showed that increasing G6PD expression in cardiomyocytes can shunt G6P to the pentose phosphate pathway, reduce G6P accumulation in the myocardium, and attenuate myocardial I/R injury [21].

#### Heart failure

In a failing heart, the predominant feature is a decreased mitochondrial oxidative capacity resulting in an inadequate energy supply. Although partially compensated by the increased production of ATP by glycolysis, it is still insufficient to maintain the normal activity of the cardiac tissue [14]. According to the current study, HK1 is more closely associated with HF. It has been reported that endothelial cell (EC) dysfunction may be an important driver of heart failure with preserved ejection fraction (HFpEF), and that promoting HK1 binding to mitochondria is a new therapeutic direction to improve heart failure. Therefore, promoting the binding of HK1 to mitochondria is a new therapeutic direction to improve heart failure. In addition, it has been claimed that long-term use of non-steroidal anti-inflammatory drugs (NSAIDs) that selectively inhibit COX2 increases the risk of heart failure, which may be associated with dysregulation of cardiac metabolism, and a significant increase in HK1 expression was found in the hearts of COX2-deficient rats, speculating that HK1 may be associated with altered cardiac energy metabolism during heart failure. However, the exact mechanism involved is unclear [48]. Therefore, it is essential to continue to explore the role of HK1 in altered energy metabolism in heart failure, which will provide potential therapeutic strategies for the treatment of HF.

In addition to HK1, the link between HK2 and cardiac hypertrophy is equally important. It has been shown that cardiac hypertrophy is associated with the accumulation of ROS and that reducing their accumulation can achieve attenuation of cardiac hypertrophy. It has been shown that in vitro overexpression of HK2 can increase the flux of the pentose phosphate pathway by increasing G6PDH activity within the pentose phosphate pathway. This was able to significantly reduce ROS accumulation and prevent ROS-induced cardiomyocyte death, with an anti-hypertrophic effect [49]. In addition, with the accumulation of ROS during cardiac hypertrophy, the level of HK2 in cardiomyocytes decreases and its dislocation from mitochondria increases. This increased dislocation may reduce angiogenesis and vascular endothelial growth factor (VEGF) levels through HIF-dependent pathways and lead to an increase in cardiomyocyte size, which in turn induces a decrease in cardiomyocyte contractility [19, 50]. In summary, stimulating an increase in HK2 levels and increasing its binding level to mitochondria may be an effective measure to alleviate myocardial hypertrophy.Several findings have been made for the use of HK in the treatment of heart failure. Current research has identified a protein phosphatase called PHLPP, which is rich in leucine and can inhibit Akt signaling by dephosphorylating it, reducing the level of HK2 bound to cardiac mitochondria and thus promoting a process associated with physiological hypertrophy, which may be beneficial in slowing down the development of pathological hypertrophy [51]. Qiangxin recipe, a well-known herbal medicine, promotes the activation of a transcription factor called Krüppel-like factor 5 (KLF5) and increases the activity of the HK2 gene promoter, which induces glucose metabolism and inhibits cardiomyocyte apoptosis, providing a novel targeted therapy for the treatment of congestive heart failure [52]. Heart failure also involves the development of vascular inflammation, and the sodium-glucose cotransporter 2 (SGLT2) inhibitor Canagliflozin (Cana) can reduce inflammation in endothelial cells via HK2 reductions, revealing that Cana has a novel anti-inflammatory mechanism via HK2 [53]. In conclusion, HKs play an important role in the development of HF, and studying its specific mechanisms and regulation may be a rewarding research direction for the treatment of HF.

### Phosphoglucose isomerase (PGI)

Phosphoglucose isomerase (PGI) is an enzyme of the second reaction step of glycolysis that reversibly isomerizes glucose-6-phosphate to fructose-6-phosphate and is widely distributed in a variety of human tissues [54]. PGI has three manifestations in cells, monomers, dimers, and tetramers. Monomers have no enzymatic activity and are usually involved in various biological pathways as cytokines. The dimer is the form of the enzyme that primarily exercises catalytic activity and consists of one large and one small structural domain, with the binding site for the glucose located at the junction between these two domains. Although the tetrameric form of PGI is also observed in vivo and in vitro, its specific function is unclear [55].

#### Heart failure

Previous studies have shown that metabolic and structural remodeling is a hallmark of heart failure and that this remodeling involves the activation of the mTOR signaling pathway. A study in adult mouse ventricular cardiomyocytes (AMVM) found that sustained inhibition of PGI induced G6P accumulation and increased G6PDH activity, redirected carbon to the pentose phosphate pathway (PPP), and led to an increase in mTOR phosphorylation and activation, ultimately contributing to increased protein synthesis in AMVM [56]. This study demonstrates that decreasing PGI activity modulates activation of the mTOR pathway, which directly affects cardiomyocyte growth. Not only that, changes in metabolic levels induced by persistent activation of mTORC1 such as reduced rates of glucose uptake and oxidation, reduced PGI activity, and increased intracellular levels of G6P were also found to precede hypertrophy [57]. In summary, heart failure and cardiac hypertrophy is associated with activation of the mTOR signaling pathway, which can be modulated by reduced PGI activity. Therefore, targeting PGI and increasing its activity may be a potential treatment for mTORC1-induced cardiac hypertrophy as well as heart failure.

#### Coronary heart disease

Glucose-6-phosphate isomerase deficiency has been shown to have a protective effect against coronary artery disease [58]. Zhang et al. found that the G6PI as a novel autoantigen was significantly increased in patients with coronary heart disease compared with healthy controls [59]. In rheumatoid arthritis, G6PI is an autoantigen that is widely present on the synovial lining surface and the small artery endothelial cell surface60. There is enough evidence to support the notion that CHD has an autoimmunity property. Currently, G6PI is being researched as an autoantigen for coronary heart disease, which differs from its glycolysis function and maybe a new indicator for the prediction of coronary heart disease.

### Phosphofructokinase-1 (PFK-1)

Phosphofructokinase 1 (PFK-1) is an important rate-limiting enzyme in glycolytic metabolism, responsible for the conversion of fructose-6-phosphate and ATP to fructose-1,6-bisphosphate and ADP. This is the third step in glycolytic metabolism. PFK-1 is a tetrameric enzyme, with three different isoforms in the human body, namely platelet isoform (PFKP), liver isoform (PFKL), and muscle isoform (PFKM). The different isoforms are mainly dependent on the cell type in which they are found [61]. PFK-1 expression is regulated intracellularly by a variety of substances, the most potent of which is fructose-2,6-bisphosphate (F-2,6-BP). In turn, the level of F-2,6-BP is regulated by fructose-2,6-bisphosphatase (PFKFB) [62]. Thus, modulation of PFKFB activity can influence PFK-1 expression through F-2,6-BP, which in turn affects metabolic fluxes of glycolysis.

#### Myocardial Infarction

Glucose metabolism accounts for only a small fraction of the energy supply in the healthy heart, but plays an important role in cardiac pathology, especially in ischemic hearts. In chronic hypoxic heart disease, elevated glycolysis leads to chronic myocardial hypertrophy. It has been found that a long-term steady increase in F-2,6-BP levels in the mouse heart can protect cardiomyocytes from hypoxic damage by increasing glycolysis. The chronically elevated levels of glycolysis may be responsible for myocardial damage in failing hearts by promoting both myocardial hypertrophy and myocardial fibrosis [63]. However, in acute hypoxic heart disease, a short period of elevated glycolysis induced by F-2,6-BP can be beneficial in reducing stress overload-induced cardiac hypertrophy. In one study, F-2,6-BP levels were significantly increased in the hearts of control mice after transverse aortic constriction (TAC) surgery, and pressure overload-induced cardiac hypertrophy, dysfunction, and fibrosis were alleviated. However, more severe hypertrophy and more severe fibrosis occurred in the hearts of transgenic mice with suppressed expression of F-2,6-BP levels [64]. The above findings suggest that PFK-2 stimulation and elevation of F-2,6-BP are critical adaptive responses to cardiac pressure overload.

MI is a hypoxia-associated myocardial injury capable of causing severe myocardial injury and cardiomyocyte apoptosis. It was found that hypoxia treatment was able to alleviate hypoxia-induced cardiomyocyte apoptosis by significantly upregulating PFKFB2 expression and activation in MI cardiomyocytes through activation of the HIF-1 / AKT axis [65]. This provides a potential therapeutic target for hypoxia-associated myocardial injury. In addition, cardiac remodeling after MI is an important cause of death, which is associated with inflammation and oxidative stress. Jaboticaba, a fruit native to Brazil, has been reported to improve the remodeling process after MI by combating inflammation and reducing oxidative stress. This process involves alterations in energy metabolism, which attenuates the activity of glycolysis-related enzymes such as LDH and PFK at higher levels resulting from MI and may in this way improve mitochondrial function [66]. In summary, increasing PFK expression and activation in the acute phase can alleviate hypoxia-induced myocardial injury, whereas attenuating PFK activity after myocardial infarction improves the remodeling process after myocardial infarction, which is of positive significance in the treatment of myocardial infarction.

#### Heart failure

An important feature of heart failure is the increased rate of glucose uptake and glycolysis, and glycolytic metabolism in endothelial cells (CM) is important for angiogenesis and pathological remodeling of the heart [67]. In a recent study, by analyzing gene expression profiles of hypertrophic cardiomyocytes, the mRNA expression level of PFKP, but not muscle isoform PFKM and liver isoform PFKL, was found to be significantly elevated in pathologically remodeled CM. Inhibition of PFKP was able to block the expression of Nppb, a marker of CM remodeling and heart failure [68]. This suggests that PFKP is involved in the stress response in CM and is a new heart failure-inducible gene. Not only that, the increase in PFK activity also further increases glycolytic flux, resulting in the accumulation of pyruvate and lactate, which leads to cardiac contractile dysfunction. In summary, elevated PKFP activity and expression are important factors contributing to heart failure, and inhibition of PFKP alleviates the stress response of CM and attenuates cardiac rational remodeling.

In addition, p53-induced knockdown of the regulator of glycolysis and apoptosis (TIGAR) improves endothelial angiogenesis by increasing glycolytic flux in the CM, leading to increased coronary capillary density and vascular function, as well as amelioration of heart failure [69]. In TIGAR KO subjected to TAC, the activity and level of the key glycolytic enzyme PFK-1 were found to be significantly increased, and pressure overload-induced diastolic dysfunction and interstitial fibrosis were ameliorated [70]. Similarly, upregulation of PFK-1 activity was found in mice with acetylation-deficient p53 4KR [71]. It has previously been shown that p53 4KR improves EC glycolytic function and mitochondrial respiration and can delay the progression of heart failure [71]. However, the protective role of PFK-1 in diastolic heart failure is not known. It is speculated that increasing the activity of PFK-1 may be a new target for the treatment of diastolic heart failure.

#### Diabetic cardiomyopathy

Diabetic cardiomyopathy (DCM) is a disease caused by disruption of the fatty acid/glucose balance due to a rise in lipid metabolism and a fall in glucose metabolism in cardiomyocytes. Under normal conditions, after feeding, insulin signaling can phosphorylate PFK-2 through the Akt and/or PKA signaling pathways, increasing the concentration of F-2,6-BP in cardiomyocytes. In this way, it promotes PFK-1 activity, increases glycolysis, and maintains the dynamic balance of fatty acid/glucose metabolism. However, in diabetic patients, glucose uptake is reduced due to a lack of insulin signaling, and PFK-2 is lysosomal-mediated degraded in the absence of insulin signaling [72]. Inhibition of PFK-1 activity and decreased glucose use derive the heart’s energy almost exclusively from lipids, which causes myocardial lipid accumulation, lipotoxicity, and mitochondrial dysfunction, ultimately leading to diabetic cardiomyopathy [72]. Not only that but it has also been found in diabetic patients that inhibition of PFK-1 activity is followed by upregulation of its upstream glycolytic intermediates. These included G6P and F6P, whose concentrations increased twofold and threefold, respectively. G6P is a direct substrate for glycogen synthesis, and its increase correspondingly increased the amount of glycogen in cardiomyocytes. F6P, on the other hand, is the first substrate of the hexosamine pathway, which also directly contributes to the rise in hexosamine pathway flux, resulting in a twofold increase in its end product, UDP-GlcNAc. The elevated UDP-GlcNAc may further contribute to the development of insulin resistance (IR) [73]. IR is a risk factor for type 2 diabetes mellitus (T2DM), and IR in hyperinsulinemia, hypertension, and hyperlipidemia can lead to T2DM. There are multiple and complex associations between IR and dyslipidemia, hypertension, and atherosclerosis, which can significantly increase the risk of developing cardiovascular disease [74]. Low-intensity exercise has been reported to prevent cardiac IR induced by a fructose-rich diet. The report, in the course of studies in male and ovariectomized rats, found that low-intensity exercise prevented the reduction of GSK-3β phosphorylation levels and enhanced GLUT4 expression in fructose-rich diet-induced hearts of males and prevented the reduction of PFK-2 phosphorylation in ovariectomized female rats. These effects favored glucose use by cardiomyocytes [75]. In summary, the presence of insulin signaling is necessary for this process, and increasing the expression and activity of PFK-1 and PFK-2 increases glucose utilization and improves cardiac function in cardiomyocytes from DCM patients.

### Aldolase

Aldolase A (ALDOA) is a reactive enzyme in the fourth step of glycolysis that breaks and converts fructose-1,6-bisphosphate (F-1,6-BP) to glyceraldehyde-3-phosphate (G3P) and dihydroxyacetone phosphate (DHAP). In vertebrates, there are three isoforms of members of the aldolase family involved in metabolism and glycolysis: aldolase A (ALDOA), the muscle and erythrocyte isoforms; aldolase B (ALDOB), the liver, kidney, and intestine isoforms; and aldolase C (ALDOC), the brain and nervous system isoforms [76]. ALDOA and ALDOC are primarily involved in glycolysis, whereas ALODB is involved in glycolysis and gluconeogenesis. Shortly after birth, the aldolase A gene (pM) is expressed primarily induced by the glycolysis of fast muscles (also known as type II fibers, which are the fibers responsible for explosive movements) in the muscles of the adult body. Later in development, pM is specifically upregulated in body muscles [77].

In addition to its involvement in carbohydrate metabolism, aldolase can function as a non-glycolytic enzyme in combination with a variety of proteins (cytoskeletal proteins, F-actin, α-microtubulin, etc.). The nuclear localization of ALDOA may influence the rate of cell proliferative activity by entering the S phase of the cell cycle, and its binding to skeletal muscle cytoskeletal proteins may be involved in the coordination of peripheral membrane transport and cytoskeletal dynamics [78, 79].

#### Myocardial infarction

MI is a myocardial necrotic disease due to persistent ischemia and hypoxia, and the occurrence of oxidative stress exacerbates this myocardial necrotic apoptosis. Some researchers claimed that low levels of ALDOA expression were found in blood samples from MI patients and hypoxia/reperfusion (H/R)-induced H9c2, and that overexpression of ALDOA slowed H/R-induced oxidative stress and apoptosis. The study noted that overexpression of ALDOA triggered the Notch 1 pathway by upregulating VEGF. Activation of this pathway reduces the accumulation of ROS, malondialdehyde in cardiomyocytes and decreases superoxide dismutase (SOD) activity, thereby protecting cardiomyocytes from H/R-induced apoptosis and oxidative stress [80]. This suggests that increasing the expression level of ALDOA during MI and promoting the upregulation of the VEGF/Notch 1/Jagg1 pathway by ALDOA is beneficial for the treatment of MI.

#### Heart failure and cardiac hypertrophy

During HF, cardiomyocyte metabolism shifts from fatty acid oxidation to glycolysis [81]. During this period, the glycolysis-associated enzyme ALDOA is overexpressed in cardiomyocytes and inhibits the activation of AMP-dependent protein kinase (AMPK) signaling in both a liver kinase B1 (LKB1)-dependent and an AMP-independent manner. Inhibition of AMPK signaling is a key mechanism of isoprenaline-induced cardiac hypertrophy [82]. Metformin and AICAR were able to block this effect of ALDOA. Therefore, blocking the AMPK inhibitory effect of ALDOA during cardiac hypertrophy would be beneficial in mitigating the progression of cardiac hypertrophy.

#### Arrhythmia

The sinus node (SAN), a tissue formed by pacemaker cardiomyocytes (PCs) wrapped around a large number of fibroblasts and heterogeneous neoplastic connective tissue, is an important structure that generates rhythmic electrical impulses to maintain the heartbeat. Its dysfunction can lead to adverse consequences such as arrhythmia, inadequate blood supply, and even cardiac arrest. A study in SAN with rhythm failure found that almost all metabolites related to the glycolytic process were reduced and that the reduction in ALDOC expression was a key factor in the decrease in glycolytic levels in SAN [83]. This study indicates that fibroblasts can interact with PCs through integrin-dependent MAPK-E2F1 signaling, drive PC-specific expression of ALDOC, and maintain aerobic glycolysis inherent in PCs to regulate pacemaker rhythm [83]. In conclusion, the above findings suggest that energy supplementation provided by promoting ALDOC expression may restore SAN dysfunction, which provides an effective therapeutic option for the treatment of arrhythmias.

In addition, atrial fibrillation (AF) is the most common of cardiac arrhythmias, and it is the leading cause of death from heart failure, stroke, and thrombotic tethering events. The search for valuable biomarkers is important for the management of AF. Zhong et al. studied peripheral plasma samples from selected patients with nonvalvular AF and control patients and found that PF4V1, THBS1, PBP, and ALODA were upregulated in plasma expression in patients with AF by proteomic and bioinformatics analysis [84]. ALDOA may be a candidate biomarker for the identification and management of AF.

#### Serum marker

Hypertrophic cardiomyopathy (HCM) is a common inherited heart disease characterized pathologically by left ventricular hypertrophy (LVH). Clinical assessment of disease progression in HCM is imperfect, such that a large number of high-risk cases remain undetected until sudden cardiac death (SCD) occurs. Using proteomic analysis, a study identified six protein-based peptides, including the ALDOA peptide, that were significantly elevated in the plasma of patients with LVH + HCM and demonstrated that these six protein-based peptides can be used as plasma biomarkers of HCM that correlate with an estimated degree of risk for the development of SCD [85]. Another study also performed a proteomic analysis in myocardial samples from HCM patients. The results of this study indicated that the metabolism-related enzymes ALDOA, creatine kinase type M, and acid-glycerate translocase had decreased expression in cardiomyocytes from patients with HCM, accompanied by an increase in the expression of structural proteins. This suggests the presence of metabolic and structural dysregulation in HCM [86]. Therefore, we can conclude that the downregulation of ALDOA in HCM cardiomyocytes may be involved in the development of HCM and that it can be used as a plasma biomarker to assess the degree of disease risk.

Congenital heart disease (CHD) is the leading cause of death in 1-year-old infants worldwide, accounting for 25% of all congenital anomalies. Ventricular septal defect (VSD) is one of the important disease categories. A study found that platelet activation and fructose and mannose metabolism maybe two relevant pathways in VSD. The study identified significant changes in the concentrations of 10 proteins, including aldolase B (ALDOB), pre-myosin α-4 chain (TPM4), and thymosin β-4 (T4), in patients with VSD by proteomic and bioinformatic analyses, which were validated and evaluated by ELISA analysis. Among them, the concentrations of ALDOB and TPM4 increased 1.5–1.7-fold relative to the healthy group. These significantly altered proteins are involved in glucose metabolism and blood coagulation pathways, which are closely linked to the development of VSD. Among these all identified proteins, ALDOB and T4 recognized VSD with the best sensitivity, and specificity and are potential biomarkers for VSD [87]. In summary, ALDOB may play a role in the disease progression of VSD through the glucose metabolic pathway. Because of its high sensitivity and specificity, it can be a potential biomarker for VSD, which is important for screening and recognizing VSD.

**Fig. 1:**
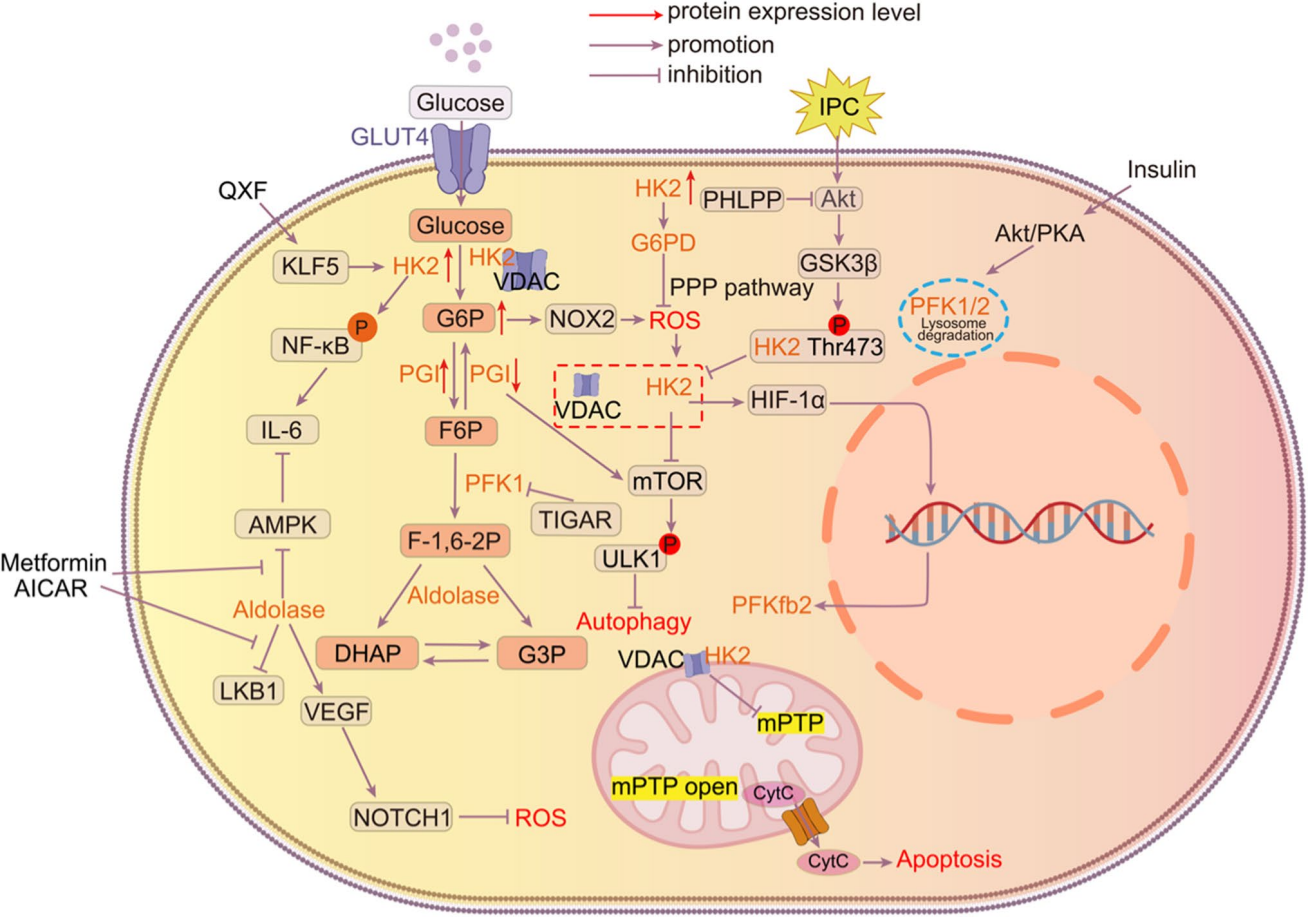
HK2 enhances NF-kB phosphorylation, promotes IL-6 production and increases vascular inflammation, ultimately leading to heart failure.G6P to accumulation can cause ROS accumulation via NOX2, promoting the separation of HK2 from VDAC.The rise of HK2 can inhibit the accumulation of ROS through the activation of the PPP bypass pathway by G6PD.The rise of HK2 can inhibit ROS accumulation through the activation of the PPP bypass pathway by G6PD. Inhibition of glycolytic flux through inhibition of PGI can promote activation of the mTOR pathway, which directly affects cardiomyocyte growth and causes myocardial hypertrophy. IPC phosphorylates HK2 at Thr-473 through the Akt signaling pathway, which increases HK2 binding to mitochondria, thereby increasing myocardial resistance to mPTP opening and cell death after reperfusion. Aldolase promotes ISO-induced cardiomyocyte hypertrophy by inhibiting AMPK activation, a process that is inhibited by metformin. Overexpression of aldolase upregulates VEGF and triggers the Notch 1 pathway to reduce ROS in cardiomyocytes.In the presence of insulin signaling, insulin signaling maintains fatty acid/metabolic homeostasis by phosphorylating PFK-2 via the Akt and/or PKA signaling pathways. The absence of insulin signaling in diabetic patients leads to disruption of this mechanism, ultimately leading to diabetic cardiomyopathy.Dissociation of HK2 from VDAC leads to opening of mPTP and release of cytochrome C into the cytoplasm, causing apoptosis

Cardiogenic shock (CS) is a state of low cardiac output with end-organ under perfusion caused by left ventricular, right ventricular, or biventricular dysfunction, with a mortality rate of up to 50%. Common causes are acute myocardial infarction (AMI), myocarditis, and acute decompensation of heart failure (HF). Both glucose and lactate have been commonly used clinically to detect CS for a century. However, they do not very much in peripheral circulating levels and do not truly aid in the diagnosis and prognosis of CS. CS4P is a newly developed proteomic risk score that incorporates hepatic fatty acid-binding protein (L-FABP), β-2-microglobulin (B2M), fructose-bisphosphate aldolase B (ALDOB), and SerpinG1 (IC1), which better refines the prediction and stratification of CS risk and is useful in the development of a risk profile for CS. and stratification [88]. ALDOB, as a newly discovered serum marker, is an important reference for the development of individualized therapeutic regimens for CS patients (Fig. 1).

### Phosphoglycerate mutase (PGAM)

Phosphoglycerol metathesis (PGAM) is a glycolytic catalase that catalyzes a reversible reaction between 3-phosphoglycerate (3-PGA) and 2-phosphoglycerate (2-PGA). During the neonatal period, the main source of energy required for cardiac metabolism gradually changes from glycolysis to fatty acid oxidation, during which PGAM expression gradually declines [89]. PGAM is a dimeric enzyme with two isoforms in mammals, the non-muscle isoform (type B) and the muscle-specific isoform (type M) [90]. Three isoforms can be composed: MM-PGAM, MB-PGAM, and BB-PGAM. BB-PGAM, named PGAM1, was originally isolated from the brain and is highly expressed primarily in the brain, kidney, and liver [90]. MM-PGAM, named PGAM2, is a muscle-specific form that is highly expressed mainly in skeletal muscle and heart. Its activity is regulated by several post-translational modifications. Among them, sumoylization is an important mechanism for regulating PGAM2 activity. The two main SUMO receptor sites are K49 and K176, and mutations in these key sites may be associated with glycogen storage disease X (GSDX) or other mutation-associated diseases [91]. MB-PGAM is predominantly expressed in the heart. The heart is the only tissue that contains all three forms of PGAM [92, 93]. In addition, PGAM5 is a specific mitochondrial serine/threonine protein phosphatase, localized to the mitochondrial membrane, which does not exert the glycolytic activity of PGAM. It plays an important role in the regulation of cell death and mitochondrial dynamics [94].

#### Ischemia–reperfusion and myocardial infarction

The current study demonstrated that necroptosis is one of the modes of death in cardiac injury under I/R conditions [95]. The major signaling pathways involved include the typical pathway RIP3-MLKL pathway as well as the atypical pathways RIP3-CaMKII-mPTP pathway, RIP3-PGAM5-Drp1-mitochondrial pathway, and RIP3-JNK-BNIP3 pathway [95]. The aim of studying these pathways is to identify the critical nodes involved in the development of I/R to help us target these sites to restore or at least minimize the myocardial damage caused by I/R. Indeed, the RIP3-MLKL pathway is the most studied mode of necroptosis induction in necroptosis, however, other atypical pathways such as the RIP3-PGAM5-Drp-1 axis are also receiving increasing attention [96]. It has been reported that a protein complex containing RIP1 and RIP3 is formed after necroptosis induction, and one of the components of this complex is PGAM5. PGAM5 has two spliceosomes, PGAM5L (long form) and PGAM5S (short form), and both play roles in the necroptosis pathway [97]. When RIP3 translocates to mitochondria, PGAM5 expression is elevated, and PGAM5S recruits the mitochondrial fission factor Drp1. It also promotes dephosphorylation of Ser637 on Drp-1, which in turn leads to mitochondrial fission, elevated ROS, and ultimately cell death. Meanwhile, PGAM5 inhibition has been shown to attenuate necroptosis and apoptosis in I/R-treated rat hearts via inhibition of Drp1, improving cardiac function and reducing inflammatory responses in an acute model of myocardial I/R [97–99]. Some researchers have claimed that while inhibition of RIP3 prevents plasma membrane rupture early during reperfusion, it does not appear to rescue cardiac dysfunction by affecting these types of necroptosis signaling but is more likely to be related to mitochondrial oxidative stress regulated by RIP3 via xanthin oxidase (XO) and manganese superoxide dismutase (MnSOD) [95]. At the subcellular level, inhibition of PGAM5 increased mitochondrial DNA copy number and transcription level, inhibited mitochondrial ROS production, and prevented aberrant opening of mPTP during I/R to achieve cardioprotective effects [100]. Taken together, PGAM5 can serve as a novel inducer of necroptosis, providing new possibilities for the treatment of myocardial infarction. In addition, since RIP3 is the confluence of these signaling pathways, reducing the nuclear translocation of RIP3 is also a new idea [95, 98]. Thus, this atypical necroptosis signaling appears to be more complex and requires further study.

#### Heart failure

A growing body of research suggests that metabolic reprogramming is an early alteration in heart failure and correlates with the severity of HF [14]. PGAM2 has the second highest level of activity in the heart after skeletal muscle [91]. An earlier study observed an approximately fivefold increase in PGAM2 protein expression in a canine model of tachycardia-induced heart failure [101]. Similarly, Li et al. detected elevated PGAM2 expression in the serum of HF patients. It also illustrates that PGAM2 is a new biomarker for assessing the severity of HF with an accuracy comparable to BNP [102]. A recent report states that sustained overexpression of PGAM2 can alter the levels of metabolites in glycolysis and the TCA cycle and may alter fatty acid and mitochondrial metabolism, which may disrupt mitochondrial function and increase cardiac stress sensitivity [103]. This predisposes the heart to severe myocardial fibrosis [64], ultimately resulting in heart failure. However, this study has not yet illustrated the mechanism by which PGAM2 overexpression affects mitochondria, which needs to be further explored. In addition, ZIKV infection significantly upregulates the expression levels of enzymes related to the glycolytic pathway, including PGAM1, and promotes cardiac fibrosis by impairing cardiac hypertrophy-associated proteins (e.g., RGS5, a GTPase activator), causing cardiac hypertrophy, which may lead to heart failure in infected patients [104].

### Enolase (ENO)

ENO is a dimeric enzyme in the glycolytic pathway that catalyzes the interconversion between 2-phosphoglycerate and phosphoenolpyruvate. Three ENO isoenzymes have been identified in higher vertebrates: α-enolase (ENO-1), β-enolase (ENO-3), and γ-enolase (ENO-2), all of which are composed of homodimers. Of these, α-enolase is universally expressed in most tissues, β-enolase is specifically expressed in muscle tissue, and γ-enolase is found mainly in neural tissue [105–107]. During development, the accumulation of these specific isoforms is often accompanied by the differentiation of two tissues with high energy requirements: αγ, γγ in the brain and αβ, ββ in the rhabdomyosarcoma [108]. γ-enolase, also called neuron-specific enolase (NSE), is expressed in neuronal tissues and neuronal tissues as an inhibitor in a wide variety of diseases (including Neuroendocrine Tumor (NET), Small Cell Lung Cancer (SCLC), Gastroenteropancreatic (GEP)-NET, etc.) biomarkers. γ-enolase also can predict adverse neurologic outcomes in comatose patients after cardiopulmonary resuscitation [109]. During cardiac development, the expression of α-enolase is significantly decreased, and the gene expression of β-enolase accounts for the second largest amount of total enolase.

In addition to its catalytic activity, ENO is involved in other physiological pathways such as growth regulation and hypoxia tolerance [107, 110]. These non-catalytic activities of ENO-1 are related to its cellular and extracellular localization. Association of ENO-1 with mitochondrial membranes is critical for mitochondrial membrane stability, whereas chelation of ENO-1 at the cell surface is essential for fibrinolytic enzyme-mediated hydrolysis of periplasmic proteins by a yet unknown mechanism [107]. As the underlying mechanisms of ENO multitasking are unclear, ENO-1-targeted therapeutic approaches need to be carefully considered in the future to avoid unwanted side effects on normal cells.

#### Myocardial infarction

Post- MI fibrosis is extremely detrimental to post-infarction repair of the heart and is an important trigger for HF. Recognition of biomarkers released from the heart in the early stages of acute myocardial infarction (AMI) is important for diagnosing myocardial ischemia and rescuing dead cardiomyocytes. The specific markers that have been applied are creatine kinase isoenzyme MB (CK-MB), cardiac troponin T (cTnT), cardiac troponin I (cTnI), and myoglobin, but they are released from the myocardium at a later stage of AMI occurrence and do not allow early ischemia diagnosis [111, 112]. To explore more valuable early biomarkers, Kurt D. Marshall et al. used H_2_O_2_ to induce necrosis in cardiomyocytes and analyzed the proteins released histologically. They found a relative increase in the number of 147 proteins including enolase αβ [113]. This has important implications for the diagnosis of ischemia–reperfusion injury. Not only that, but in a recent study it was also suggested that the serum concentration of β-enolase was significantly elevated in AMI and that the rate of rise and fall in its concentration was faster and steeper than that of CK-MB, with a higher sensitivity [114]. These findings suggest that beta-enolase is likely to be a more effective marker of early myocardial infarction.

Among the various triggers of HF, fibrosis after myocardial infarction (MI) is an important aspect. Previous studies have confirmed that TGF-β-Smad2/3 signaling in fibroblasts is a major mediator of the fibrotic response [115]. ENO was able to mediate the fibrogenic effects of TGF-β1, volatile TGF-β1-independent fibrogenesis [116]. Activation of this process is highly detrimental to remodeling after cardiac injury. To further explore the relationship between ENO and myocardial fibrosis, Jing-jing Ji et al. inhibited the transcriptional activation of ENO-1 by regulating the acetylation of Nr4a1, an acetylated protein, using tissue kinin-releasing enzyme binding protein (KS/Serpina3c). Overactivation of glycolysis during MI was found to be inhibited and fibrosis after MI was attenuated. This may be because ENO-1 inhibition antagonized the promotional effect of Serpina3c on CFs proliferation 117. In another study, inhibition of the FAK/Ras/c-myc/ENO1 pathway effectively suppressed aerobic glycolysis and ameliorated liver fibrosis [118]. The above findings suggest that the overactivation of ENO-1 has a fibrosis-promoting effect and that cardiomyocyte activation and myocardial fibrosis after MI can be alleviated by inhibiting ENO-1 [119].

#### Heart failure

Adriamycin (Dox) is one of the most effective chemotherapeutic agents against many types of cancer (e.g., acute leukemia, sarcoma). However, clinical use of Dox leads to cardiomyocyte apoptosis, decreases cardiac contractile function, and causes irreversible cardiac damage and dysfunction, which is highly correlated with mitochondrial damage [120–122]. ENO-1 is a regulator of cardiac mitochondria, which is partially located in the mitochondria of rat cardiomyocytes. When co-localized with the mitochondrial membrane, α-enolase has a stabilizing effect on the mitochondrial membrane by preventing Ca^2+^-induced mitochondrial transmembrane potential, matrix swelling, and cytochrome C release. This process is associated with VDAC1. In addition, because VDAC1 is a key regulator of the cell death pathway, alpha-enolase also has the potential to regulate apoptosis in cardiomyocytes [123]. In Dox-induced cardiomyopathy, α-enolase dissociates from mitochondria and Dox replaces α-enolase on the outer mitochondrial membrane, which results in mitochondrial dysfunction and activation of the cell death pathway [123]. Not only that, but in another study a rise in mRNA expression of α-enolase was observed, accompanied by an increase in AMPK dephosphorylation. This study also demonstrated that genetic silencing of α-enolase was able to attenuate Dox-induced apoptosis and mitochondrial dysfunction by inhibiting the release of mitochondrial CtyC into the cytoplasm, attenuating a series of Dox-induced reactions such as rapid loss of the mitochondrial electrochemical gradient and Caspase3 activation [124]. The above study illustrates that α-enolase has an independent catalytic role in inducing apoptosis and mitochondrial dysfunction in cardiomyocytes and may have some ATP deprivation effects. Promoting its binding to the outer mitochondrial membrane and inhibiting the overexpression of α-enolase could attenuate Dox-induced myocardial injury.

In addition, the role of ENO in cardiac hypertrophy has been demonstrated. During cardiac hypertrophy, compensatory elevation of α-enolase protects cardiomyocytes from pathological hypertrophy [125]. Excessive elevation of α-enolase, however, leads to an elevated ratio of α-enolase to β-enolase concentrations, and this dysregulation of the ratio may be associated with contractile dysfunction during cardiac hypertrophy [126, 127].

#### Diabetic cardiomyopathy

In the pathogenesis of diabetes, oxidation, and nitration of proteins are important contributors to diabetes [128]. Researchers found significantly elevated expression and nitration levels of α-enolase in the hearts of diabetic rats, but no significant changes in activity or oxidation levels. The study further confirmed that α-enolase is most susceptible to nitration at two sites, Tyr 257 and Tyr 131. Nitrated alpha-enolase activity is significantly decreased, which results in reduced myocardial energy stores and is an important contributor to the abnormal energy metabolism associated with diabetic cardiomyopathy, and thus secondary to diabetic cardiomyopathy [129]. Meanwhile, the upregulation of α-enolase expression may neutralize the oxidative stress caused by hyperglycemia, which is a protective mechanism for the cells [129].

### Pyruvate kinase (PK)

Pyruvate kinase (PK) is another rate-limiting enzyme in the glycolytic pathway that catalyzes the irreversible conversion of acid-enol pyruvate (PEP) to pyruvate while transferring the high-energy phosphate bond of PEP to ADP to generate ATP. In mammals, there are four isoforms of pyruvate kinase: the L-type, the R-type, the M1-type, and the M2-type. Of these, PKL is found mainly in gluconeogenic tissues, especially the liver; PKR is found mainly in erythrocytes and hematopoietic tissues.PKM1 is highly expressed as a tetramer in cardiac, skeletal muscle, and brain tissues; PKM2 is expressed as a monomer, dimer, or tetramer in the lungs, spleen, kidneys, and testes [130–132]; in mature differentiated cells, PKM1 predominates, while PKM2 is highly expressed in cancer cells and embryos [133, 134]. In addition to its glycolytic catalytic role, PKM2 can act as a transcriptional regulator or protein kinase following nuclear translocation to regulate a variety of pathways such as apoptosis, mitosis, and tumor cell growth [130, 133].

#### Myocardial infarction

After MI injury, the repair process of the heart includes three coordinated phases of remodeling of extracellular mechanisms, neoangiogenesis, and cardiomyocyte (CM) proliferation [135]. Over the past two decades, many studies have attempted to explore the regulatory mechanisms of the CM cell cycle to induce CM proliferation [135, 136]. It was found that after ischemic injury, the sustained expression of HIF-1 was able to make PKM2 expression superior to that of normally expressed PKM1 and modify PKM2 through signaling proteins and post-translational modifications, which may be beneficial to cardiomyocyte proliferation [134]. Researchers have also engaged in a lively discussion about the specific mechanisms of PKM2 in regulating the cardiomyocyte cycle. On the one hand, Ludger Hauck et al. claimed that Pkm2 can directly interact with β-connexin (Ctnnb1) in the cytoplasm of cardiomyocytes (CM), inhibit the phosphorylation of Ctnnb1 via Akt at Ser552 and Try333, prevent Ctnnb1 from translocating to the nucleus, and then inhibit the transcription of proliferation-associated target genes (such as Myc and Cyclin D1) transcription, which adversely affects cardiac repair after myocardial infarction [137]. Interestingly, however, when PKM2 translocates to the nucleus, it can interact directly with Ctnnb1 in the nucleus of cardiomyocytes and the complex cooperates with T-cell factor 4 (TCF4) to up-regulate its downstream targets, Cyclin-D1 and C-Myc, to transcriptionally induce genes encoding anti-apoptotic proteins. PKM2 was also positively regulated by C-Myc, suggesting the existence of a positive feedback loop between PKM2 and c-Myc, which contributes to the cardiomyocyte cycle and cardiac regeneration [133, 136]. Procatecholaldehyde (PCA) is one that greatly attenuates cardiac injury and protects cardiomyocytes from apoptosis through the β-linker protein/TCF4 signaling cascade [133]. The above two different studies illustrate that the role of PKM2 in cardiac repair may be related to its intracellular localization and that PKM2's nuclear translocation may be a key factor in the treatment of myocardial infarction. On the other hand, PKM2 has an enzymatic function to enhance G6pd and redirect glucose carbon flow into the PPP anabolic pathway. Elevated PPP leads to reduced ROS production and oxidative DNA damage, thereby inhibiting postnatal cardiomyocyte cycle arrest [136]. Therefore, further investigation of the regulators in the two key pathways of PKM2-induced CM proliferation may be a potential therapeutic approach.

In addition, inflammation is a key factor in MI injury. In the hearts of patients with non-ST-segment elevation myocardial infarction myocardial infarction, nuclear translocation of PKM2 acts as a transcriptional regulator of pro-inflammatory genes and promotes transcription of cellular pro-inflammatory factors (e.g., IL6, IL-1β, and IFNγ), which cause myocardial injury [138]. Iminostilbene can target PKM2 to reduce macrophage inflammation, thereby significantly attenuating MI/R injury [139]. Overall, the intracellular localization of PKM2 is a key factor in its differential effects. Using PKM2 as a therapeutic target to promote the transcription of proliferative genes and inhibit the release of pro-inflammatory factors is an important strategy for the treatment of myocardial infarction.

#### Heart failure

Normally, the adult heart exhibits high levels of PKM1 and low levels of PKM2.PKM1 plays a critical role in maintaining the cardiac homeostatic response to hemodynamic stress [140]. PKM1 is reduced and PKM2 is elevated during the onset of heart failure. PKM1 deficiency inhibits pyruvate dehydrogenase (PDH) activity by reducing the production of its product, pyruvate, which reduces TCA flux and impairs mitochondrial energy production, and exacerbates pressure overload-induced cardiac insufficiency and fibrosis, whereas induced PKM1 overexpression protects the heart from systolic dysfunction and is critical for maintaining glucose uptake and glycolysis for ATP production and macromolecular biosynthesis [140].

In contrast to PKM1's protective effect on the heart, PKM2 is a deleterious factor in pathological cardiac remodeling. Myocardial fibrosis is an important pathological process in hypertension-induced cardiac remodeling. Among them, the TGF-β / Smad2 / 3 pathway and the Jak2 / Stat3 signaling pathway are the main pathways for fibrogenesis [141]. It has been demonstrated that PKM2 can exacerbate cardiac fibrosis by activating these two pathways. In addition, PKM2 also exacerbates Ang II-induced cardiac fibrosis by stimulating oxidative stress [142], and inhibition of PKM2 significantly reduced cardiac fibroblast proliferation, migration, and collagen synthesis in vitro [142]. Therefore, we can conclude that negative regulation of PKM2 may improve cardiac remodeling in hypertension by inhibiting cardiac fibrosis.

In addition, right ventricular fibrosis in patients with type 2 pulmonary hypertension (PH) leads to right ventricular (RV) failure, which is the most common cause of death in patients with PH [143]. The pathophysiologic basis of RV failure is complex and multifactorial, in which CM dysfunction is a major determinant of RV failure [144]. Overactivated poly (ADP-ribose) polymerase 1 (PARP1) promotes PKM2 expression and nuclear translocation, increases glycolytic gene expression, nuclear translocation of NF-kB, and expression of proinflammatory factors, resulting in CM dysfunction [144]. Elevated PKM2 expression is also associated with impaired right ventricular function and the development of right ventricular fibrosis [145]. Inhibition of PKM2 and restoration of a normal PKM2/PKM1 ratio effectively reduces PH [146]. Taken together, PKM2 has pleiotropic effects targeting the RV and pulmonary vascular system, and the inhibition of PKM2 expression and nuclear translocation may be an effective strategy for the treatment of RV failure.

Many chemotherapeutic agents (including anthracyclines, such as doxorubicin) can promote heart failure by non-specifically inducing the pro-apoptotic transcription factor p53 in the heart [147]. In the failing heart, tetrameric PKM2 binds directly to p53, inhibiting p53 transcriptional activity and apoptosis in the high oxidized state, but enhancing in the low oxidized state, where the redox state of cysteine-423 of tetrameric PKM2 is critical for the differential regulation of p53 transcriptional activity [147]. Based on existing reports, the small molecules TEPP-46 and 2-DG can promote the formation of stable tetramers with high pyruvate kinase activity [148], providing new ideas for the treatment of chemotherapeutic drug-induced heart failure.

To date, heart transplantation is the most effective treatment for end-stage heart failure. However, chronic and acute rejection is the greatest cause of postoperative mortality in patients. In a study, PKM2 was found to be widely expressed in post-transplant cardiac tissues but not in T cells and other immune response cells [149]. Suggests that PKM2 may regulate cardiomyocyte survival in acute rejection.

#### Cardiomyopathy

PKM2 is usually absent in healthy adult cardiomyocytes but elevated in cardiomyopathies, where PKM2 is usually present in the heart as an inactive dimer. Tang et al. found that Jmjd4 interacts with Hsp70 to mediate the degradation of PKM2, which is dependent on hydroxylation of the PKM2 K62 site by Jmjd4. In idiopathic and familial DCM, Jmjd4 expression is significantly reduced in the hearts of patients, leading to PKM2 accumulation [150]. Tang et al. used the small molecule activator TEPP-46 to convert PKM2 dimers to enzymatically active PKM2 tetramers and found that this was able to reduce TCA-induced metabolic impairments and rescue the metabolic dysfunction and cardiac hypertrophy induced by knockdown of Jmjd4. The function of PKM2 in cardiomyocytes has not yet been thoroughly analyzed but this study at least partially finds the positive role of elevated PKM2 tetramers in DCM.

Sepsis is a systemic inflammatory response syndrome caused by gram-negative bacteria, often accompanied by multiple organ dysfunctions. Sepsis-induced cardiomyopathy (SIC) is one of the most serious complications, capable of inducing adverse cardiomyocyte apoptosis, mitochondrial abnormalities, and oxidative stress, which ultimately impairs myocardial function [151]. PKM2 deficiency exacerbates calcium homeostasis by impairing sarcoplasmic/endoplasmic reticulum calcium ATPase 2a (SERCA2a) expression, resulting in lipopolysaccharide (LPS)-induced cardiac dysfunction [151]. PKM2 plays a critical role in Gram-negative sepsis-induced cardiomyopathy and provides an attractive target for the prevention and treatment of septic cardiomyopathy.

Dagliflozin (DAPA) is a drug for the treatment of diabetes, and studies claim that it is also protective against cardiomyopathy caused by cardiorenal syndrome (CRS). In the context of CRS, PKM2 expression is reduced, and mitochondrial structure is damaged and dysfunctional.DAPA can complement PKM2 expression and allows it to interact directly with protein kinase 1 (PP1) and FUNDC1, activating FUNDC1 in a dephosphorylated manner. It promotes FUNDC1-dependent mitochondrial autophagy and attenuates mitochondrial damage and defects, resulting in protecting myocardial structure and cardiac function [152].

#### Atherosclerosis

PKM2 has been demonstrated to be upregulated in monocytes/macrophages from patients with atherosclerotic coronary artery disease^153^, enhancing macrophage accumulation in atherosclerotic lesions primarily by promoting foam cell formation and exacerbating inflammatory responses [154]. Genetic deletion of PKM2 or restriction of its nuclear translocation in macrophages was found to alleviate the atherosclerotic lesions by inhibiting inflammation and enhancing erythropoiesis [155]. Unexpectedly, deletion of PKM2 in macrophages increased the expression of LRP (LDLR-related protein)-1, which may mitigate the progression of atherosclerosis by modulating the macrophage inflammatory response in the microenvironment of atherosclerotic lesions [155]. However, the exact mechanism of how PKM2 regulates LRP-1 is unclear and will remain an area for future research. In addition, nuclear PKM2 can activate STAT3 and drive the transcription of pro-inflammatory genes IL-6 and IL-1β in a pSTAT3-dependent manner, exacerbating the inflammatory response [156]. Not only that, but PKM2 is also able to mediate the activation of the NLRP3 inflammatory vesicle/stress granule design in macrophages and the subsequent release of pro-inflammatory mediators, such as IL-18 and IL-1β [148], induces vascular smooth muscle cell proliferation and migration and increases plaque vulnerability and through upregulation of PKM2-dependent glycolysis [157]. The above findings suggest that the glucose-ROS-PKM2-STAT3 axis and the search for PKM2 inhibitors are new directions for anti-inflammatory interventions in cardiovascular disease.

### Lactatedehydrogenase (LD or LDH)

Lactate dehydrogenase (LD or LDH) is a tetrameric enzyme that catalyzes the redox reaction between pyruvate and L-lactate and is one of the key enzymes of glycolysis. In mammals, LDH has three subunits, LDHA, LDHB, and LDHC, which can constitute six tetrameric isoenzymes. Of these, LDHA is found mainly in skeletal muscle and liver, and is also known as the M subunit; LDHB is found mainly in the myocardium, brain, kidney, and erythrocytes [158]. LDHA and LDHB can form homo- or heterotetramers LDH1-5 (LDH1, LDH2, LDH3, LDH4, and LDH5), which are expressed predominantly in the cytoplasm [159]. Different isoenzymes have different catalytic roles. LDHA catalyzes the conversion of pyruvate to lactate, while LDHB catalyzes the conversion of lactate to pyruvate [159]. LDH6 is composed of homologous LDHC (LDH-C4), which is found primarily in human testes and spermatozoa and is associated with male fertility [159].

#### Myocardial infarction

Control of metabolic conversion is an important factor in cardiac repair after myocardial infarction and can effectively mitigate the loss of regenerative capacity in the mammalian heart [160]. One study found that overexpression of LDHA induced metabolic reprogramming, stimulating CM proliferation by alleviating ROS and inducing M2 macrophage polarization [160], facilitating cardiac remodeling, suggesting that LDHA may be an effective target to promote cardiac repair after myocardial infarction [160].

#### Heart failure

Cardiac hypertrophy is an enlargement of the myocardium due to overload stress and is a major cause of heart failure [161]. Metabolic remodeling is an early event in this process [57, 162]. Cardiac pressure overload can significantly upregulate LDHA expression in the heart, and LDHA deficiency in cardiomyocytes can lead to defective cardiac hypertrophy and heart failure. In contrast, lactate can stimulate ERK (extracellular signal-regulated kinase) expression by stabilizing NDRG3 (N-myc downstream-regulated gene 3) to rescue growth defects caused by LDHA knockdown [162]. The above findings emphasize the importance of the LDHA/lactate/NNDRG3 axis in the heart in controlling cardiac hypertrophic growth induced by elevated blood pressure [162]. Furthermore, LDHB plays an important role in the treatment of Ang II-induced cardiomyocyte hypertrophy. A miR-375-3p inhibitor has been found to inhibit Ang II-induced cardiomyocyte hypertrophy by promoting LDHB expression [161]. Yamaguchi et al. found that serum LDH may also be an important predictor of 90-, 180- and 365-day all-cause mortality in patients with acute decompensated heart failure, suggesting that serum LDH has important prognostic value in acute decompensated heart failure [163].

#### Aortic constriction

**Fig. 2:**
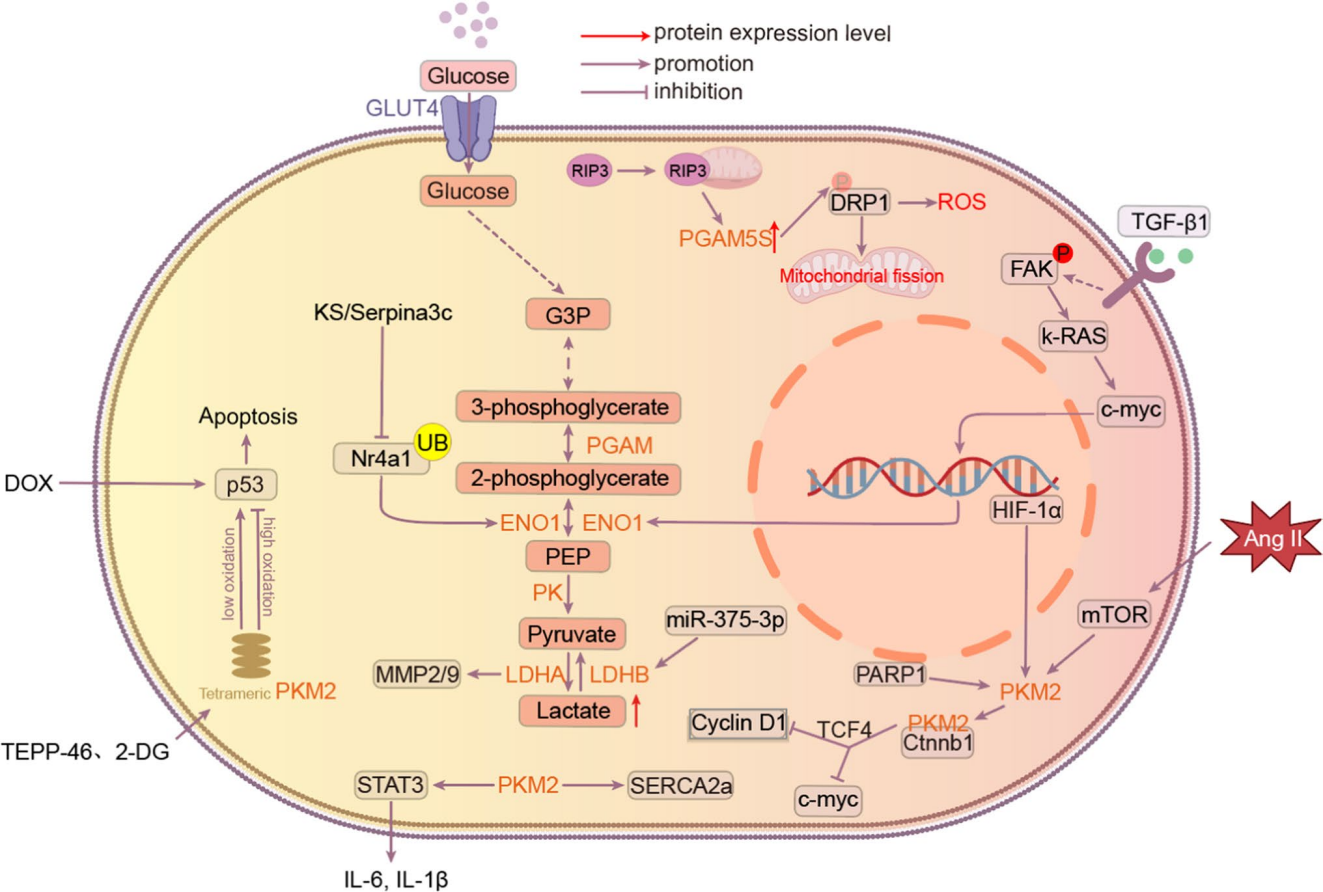
KS/Serpina3c inhibits transcriptional activation of ENO1 by regulating the acetylation of Nr4a1, thereby reducing glycolytic overactivation to prevent fibrosis after ischemia/reperfusion injury. In the failing heart, PKM2 tetramers bind directly to p53 and inhibit p53 transcriptional activity and apoptosis in the high oxidative state, thereby alleviating the progression of heart failure. However, they are enhanced in the low-oxidized state, and the small molecules TEPP-46 and 2-DG can promote PKM2 tetramer formation. When RIP3 translocates to mitochondria, it induces elevated PGAM5S expression, promotes Ser637 dephosphorylation on Drp-1, and facilitates mitochondrial fission. Inhibition of the FAK / Ras / c-myc / ENO1 pathway effectively suppressed aerobic glycolysis and ameliorated hepatic fibrosis.Pkm2 directly interacts with β-linker protein (Ctnnb1) in the cytoplasm of cardiomyocytes (CM), preventing translocation of Ctnnb1 to the nucleus, and subsequently repressing proliferation-related target genes, such as Myc and Cyclin D1). When Pkm2 translocates to the nucleus, it can directly interact with Ctnnb1 in the nucleus of cardiomyocytes to form a complex that cooperates with T-cell factor 4 (TCF4), up-regulates its downstream targets Cy-clin-D1 and C-Myc, and transcriptionally induces genes encoding anti-apoptotic proteins.PKM2 exacerbates proliferation-related genes through activation of the TGF-β / Smad2 / 3 pathway and the Jak2 / Stat3 signaling pathways to exacerbate fibrosis

Aortic dissection (AD) is a disease with a high mortality rate and a lack of effective drug therapy. Recent studies have suggested that AD progression may be closely linked to glucose metabolism. LDHA is a key enzyme in the glycolytic pathway, and researchers have found that in the presence of hyperglycemia, increased LDHA promotes the production of MMP2/9, stimulates extracellular matrix (ECM) degradation, and facilitates the transition of human aortic vascular smooth muscle cells from contractile to synthetic phenotype to attenuate AD progression. At the same time, the upregulation of lactate, a product of LDHA, was also able to stabilize and promote the growth and phenotypic transformation of cardiomyocytes and VSMC [164]. Therefore, we hypothesized that LDHA and its product lactate may be therapeutic targets for AD (Fig. 2) (Table 1).

**Table 1:** Selected treatments related to glycolytic enzymes and their mechanisms

Enzymes	Related diseases	Treatment	Mechanism	Effects	References
Hexokinase	Myocardial infarction	IPC	Phosphorylation of HK2 at Thr-473 via the Akt signaling pathway, thereby increasing HK2 binding to mitochondria	Improved myocardial resistance to mPTP openig and cell death after reperfusion	[16]
Hexokinase	Myocardial infarction	Low temperature	Similar to IPC	Reduces cardiac energy demand and prevents ischemia–reperfusion injury	[37]
Hexokinase	Cardiomegaly	PHLPP	Dephosphorylates Akt and reduces the level of HK2 bound to cardiac mitochondria	Associated with the development of cardiac hypertrophy	[51]
Hexokinase	Ischemia–reperfusion	Resveratrol	Inhibits VDAC1 phosphorylation via the Akt-GSK3β pathway and promotes de-VDAC1 binding to HK2	Prevention of ischemia–reperfusion injury	[32]
Hexokinase	Ischemia–reperfusion	Melatonin	It inhibits mitochondrial fission by activating AMPKα	Protects the cardiac microvascular system from IR	[38]
Hexokinase	Ischemia–reperfusion	TAT-HK2	Induced HK2 translocation from mitochondria	Exacerbates cardiac reperfusion injury	[41]
Hexokinase	Heart failure	QXF	Promote the activation of KLF5, which in turn increases the activity of the HK2 gene promoter	Induces glucose metabolism and inhibits cardiomyocyte apoptosis	[52]
Hexokinase	Heart Failure	Cana	Inhibition of IL-6 release and ERK1/2 phosphorylation by reducing HK2 expression, ultimately leading to reduced inflammation in endothelial cells	Reducing Vascular Inflammation in Heart Failure	[53]
PFK1	Heart failure	TIGAR	Increases endothelial glycolytic function	Improves endothelial angiogenesis and thereby improves heart failure	[69]
Aldolase	Heart Failure	Ginsenoside Rg1	It regulates the aldolase/AMP-activated protein kinase/PINK1 pathway	Limiting nutritional stress-induced H9c2 cell injury	[201]
ENO	Heart failure	KS/Serpina3c	Inhibits transcriptional activation of ENO1 by regulating acetylation of Nr4a1, ultimately reducing excessive activation of glycolysis	Prevention of myocardial fibrosis after MI	[117]
PKM	Myocardial infarction	PCA	Promoting Cy-clin-D1 and C-Myc expression through regulation of the β-linked protein/TCF4 signaling cascade	Reduces heart damage and protects cardiomyocytes from apoptosis	[202]
PKM	Ischemia–reperfusion	ISB	Targeting PKM2 to reduce macrophage inflammation	Reduces damage caused by I / R	[139]
PKM	Heart failure	TEPP-46、2-DG	Promotes the formation of stable tetramers with high pyruvate kinase activity	It provides a new idea for the treatment of chemotherapy drug-induced heart failure	[148]
LDH	Cardiomegaly	miR-375-3p inhibitors	Induced LDHB expression	It inhibits Ang II-induced cardiomyocyte hypertrophy	[161]

## Glycolytic bypass pathway and cardiovascular disease

### Polyol pathway

The polyol pathway is the process of oxidative reduction of glucose to fructose, which involves two key enzymes, aldose reductase (AR) and sorbitol dehydrogenase (SDH). Of these, AR reduces glucose to sorbitol while its cofactor, NADPH, is oxidized to NADP.SDH oxidizes sorbitol to fructose while reducing NAD to NADH [12, 165]. This pathway is thought to be strongly implicated in diabetic and nondiabetic myocardial ischemic injury, primarily by causing cellular oxidative stress and late AGEs (end products of glycosylation) formation to exacerbate ischemic myocardial injury [166, 167]. Under normal conditions, only about 3% of glucose enters the polyol pathway, whereas in hyperglycemia, the glucose entering the polyol pathway rises to more than 30% [168]. Activation of the polyol pathway during I/R induces oxidative stress through three main pathways. First, elevated ROS levels during ischemia increase AR activity, leading to an increase in the cytoplasmic NADH / NAD + ratio, which further induces the expression of HIF-1α and the transcription of the transferrin receptor (TfR) gene, triggering ATP depletion and tissue damage [169]. In addition, the clearance of ROS requires the involvement of reduced glutathione (GSH), a cofactor of glutathione reductase (GR), and its depletion leads to a decrease in the level of reduced GSH, which prevents the clearance of ROS and exacerbates the oxidative stress injury [165]. Oxidative stress may further lead to the inactivation of SERCA, allowing for reduced Ca^2+^ reuptake in the sarcoplasmic reticulum and myocardial contractile dysfunction [170]. Second, overactivation of the polyol pathway accumulates excess NADH in the second step, which is a substrate for NADH oxidase and can lead to the production of more superoxide anions [168]. Finally, fructose produced by the polyol pathway can be further metabolized into fructose-3-phosphate and 3-deoxyglucosone, increasing the formation of AGEs [168]. Therefore, the novel therapy of protection against ischemic cardiomyopathy through the inhibitory effect of polyol or aldose reductase pathways has attracted interest. Several studies have demonstrated that the inhibitory effect of increased NADH/NAD + ratio and oxidative stress injury in the heart through AR-induced increases in polyol pathway flux protects both diabetic and nondiabetic myocardium from ischemia–reperfusion injury [169, 171–174]. In addition, recent studies have found that elevated myocardial fructose and SDH may be associated with diabetic patients with diastolic dysfunction. Fructose exacerbates the lipotoxicity of diabetic cardiomyopathy by promoting the formation of cytoplasmic lipid inclusion bodies in cardiomyocytes, and the inhibition of SDH protects the ischemic myocardium and alleviates diastolic dysfunction [167, 175].

### Hexosamine biosynthetic pathway (HBP)

The hexosamine biosynthesis pathway (HBP) is another ancillary pathway of glycolysis capable of converting fructose-6-phosphate (F-6-P) and glutamine to glucosamine-6-phosphate (GlcN-6P) via glutamine-fructose-6-phosphate transaminase (GFPT), and ultimately synthesizing riboside diphosphate N-acetylglucosamine (UDP-GlcNAc). There are two isoforms in humans, GFPT1 and GFPT2, with GFPT2 being the main type in the heart [176]. UDP-GlcNAc is a substrate for a variety of biosynthetic pathways such as proteoglycans, hyaluronic acid, and glycolipids [12]. It also serves as a substrate for O-GlcNAc transferase (OGT) to O-GlcNAcylate proteins, which regulates cellular functions such as cell survival, signaling, and protein stability, and is thought to prevent cell death in response to stress [176–178]. It has been found that increased post-translational O-GlcNA acylation due to HBP activation may be associated with systolic and diastolic dysfunction in diabetic cardiomyopathy [179]. In addition, oxidative stress is an important risk factor in a variety of cardiovascular diseases, including diabetic cardiomyopathy, myocardial infarction, and heart failure. Oxidative stress has been reported to inhibit catalytic enzymes of the upstream pathway of glycolysis, including hexokinase, glyceraldehyde-3-phosphate dehydrogenase, and PFK, resulting in the accumulation of upstream intermediates (e.g., F-6-P) and shunting of glucose-carbon fluxes to the HBP [180]. Increased fluxes of HBP play a dual role. Acute upregulation of HBP is cardioprotective. Zhang et al. found that nuclear Tisp40, a membrane-resident transmembrane protein enriched in cardiomyocytes that is cleaved and released into the nucleus in response to ER stress, promotes HBP flux and protein O-GlcNAcylation by binding to the promoter of GFPT1, and is capable of attenuating myocardial injury in the ischemic heart [176]. Chronic activation, however, can cause protein dysfunction through sustained elevation of protein O-GlcNAcylation, which ultimately leads to cardiovascular diseases such as diabetic cardiomyopathy, cardiac hypertrophy, ischemic cardiomyopathy, and heart failure [180]. Tran et al. found that GFPT1 overexpression under hemodynamic stress caused upregulation of HBP, which subsequently induced heart failure and cardiac remodeling through persistent chronic activation of mTOR [181]. U Rajamani et al. found that in diabetic patients, hyperglycemia activates HBP and leads to reduced BAD phosphorylation and BAD-Bcl2 dimer formation and accumulation, which mediates HBP-induced cardiomyocyte apoptosis and may be associated with myocardial contractile dysfunction during episodes of type 2 diabetes [182].

### Pentose phosphate pathway (PPP) and single-carbon metabolic pathways

The single-carbon metabolic pathway and the PPP pathway are the two main pathways for NADPH production in vivo. The activity of glucose-6-phosphate dehydrogenase (G6PD or G6PDH), the key rate-limiting enzyme of the PPP pathway, increases in response to oxidative stress stimulation, and the PPP pathway is up-regulated in response to stress overload, with some compensatory effects in early life [183, 184]. In a study, it was noted that in the case of pressure overload-induced heart failure, there is a significant elevation of cardiac ROS, depletion of antioxidant defense mechanisms, and a decrease in the levels of NADPH (the major antioxidant cofactor) and GSH production [184]. It also indicates that ATF4 (a transcription factor) can maintain NADPH homeostasis and cardiac function by directly controlling the expression of genes in the single-carbon metabolic pathway and the PPP, and has cardioprotective effects [184]. In addition, Takao Kato et al. demonstrated that dichloroacetate improved CHF by increasing NADPH and GSH levels by activating the PPP and enhancing G6PD activity [183]. In conclusion, activation of the PPP pathway and the single-carbon metabolic pathway attenuates oxidative stress in the myocardium and contributes to the improvement of HF.

In ischemic heart disease, G6PD is required to maintain cellular GSH levels and prevent ischemia–reperfusion-induced myocardial injury [185]. HBP and PPP can be tightly coupled through the O-GlcNAcylation of G6PD. Ou et al. found that hypoxic adaptation can further activate G6PD by using relevant inflammatory cytokines (IL-6、IL-1β) to increase O-GlcNAcylation in the heart and activate the HBP pathway. This improves the PPP pathway enhances redox homeostasis and attenuates cardiac I/R injury [186]. Thus, O-GlcNA acylation of G6PD is promising as a new therapeutic target for ischemic heart disease. In addition, the PPP pathway was also found to be active during acute episodes of cardiac ischemia–reperfusion, and inhibition of PPP oxidation by ischemic preconditioning was able to reduce creatine kinase release and protect the heart from ischemic injury [187].

PPP may also be involved in processes such as myocardial repair in patients with coronary heart disease and diabetes [188, 189]. Recently, a study has found that PPP can act as a novel oxygen sensor and regulate hypoxic coronary artery diastole by modulating the activity of the SERCA to reduce intracellular calcium concentration. However, whether this novel function works under various physiological and pathological conditions needs further investigation [189]. In addition, the researchers found from cardiac progenitor cells (CPCs) of diabetic mice that key activities of the PPP pathway, G6PD, or transketolase were reduced and apoptosis was activated. Re-PPP pathway using benfotiamine was able to rescue these CPCs [188]. This indicates that the PPP pathway's activation may be a new therapeutic target to promote myocardial repair in diabetic patients.

### Glycogen metabolism

Glycogen, an important endogenous storage form of glucose in the body, contributes significantly to overall cardiac energy production, accounting for 41% of ATP production from glucose metabolism [190]. In normal and hypertrophied hearts, glucose from glycogen is preferentially oxidized relative to exogenous glucose. This maximizes ATP production, reduces H^+^ production, and decreases Ca^2+^ overload [190–192]. Calcium overload may be an early event in LV dysfunction during reperfusion [192]. Previous studies demonstrated that fasting protects the heart from ischemic injury by increasing glycogen utilization during ischemia [193]. More recently, Mohamed et al. found that inhibition of GSK-3 during reperfusion partially allocates glucose-6-phosphate to the glycogen synthesis pathway, decreases the rate of glycolysis, reduces H^+^ production and intracellular acidosis, and decreases Ca^2+^ overload. This limits LV dysfunction in early reperfusion injury, contributes to improved mitochondrial function and cell viability, and reduces infarct size [192]. Similarly, ischemic preconditioning ameliorates myocardial ischemia by reducing the accumulation of glycolytic catabolic products by inhibiting glycogenolysis during sustained ischemia [194].

Glycogen metabolism also has an important role in cardiac hypertrophy. It has been found that the overall rate of myocardial glycolysis increases in hypertrophied hearts during aerobic perfusion, but not during low-flow ischemia [195]. Glycogen is an important source of glucose during low-flow ischemia, accounting for a significant percentage of the total rate of glycolysis. Not only that but the rate of glycogen renewal (simultaneous synthesis and degradation) is accelerated during severe low-flow ischemia [195, 196]. D Mancini et al. showed that increasing the proportion of carbohydrates in the diet of patients with CHF exhaustion slowed the utilization of glycogen stores and improved exercise tolerance in CHF patients [197].

### Serine biosynthetic pathway

**Fig. 3:**
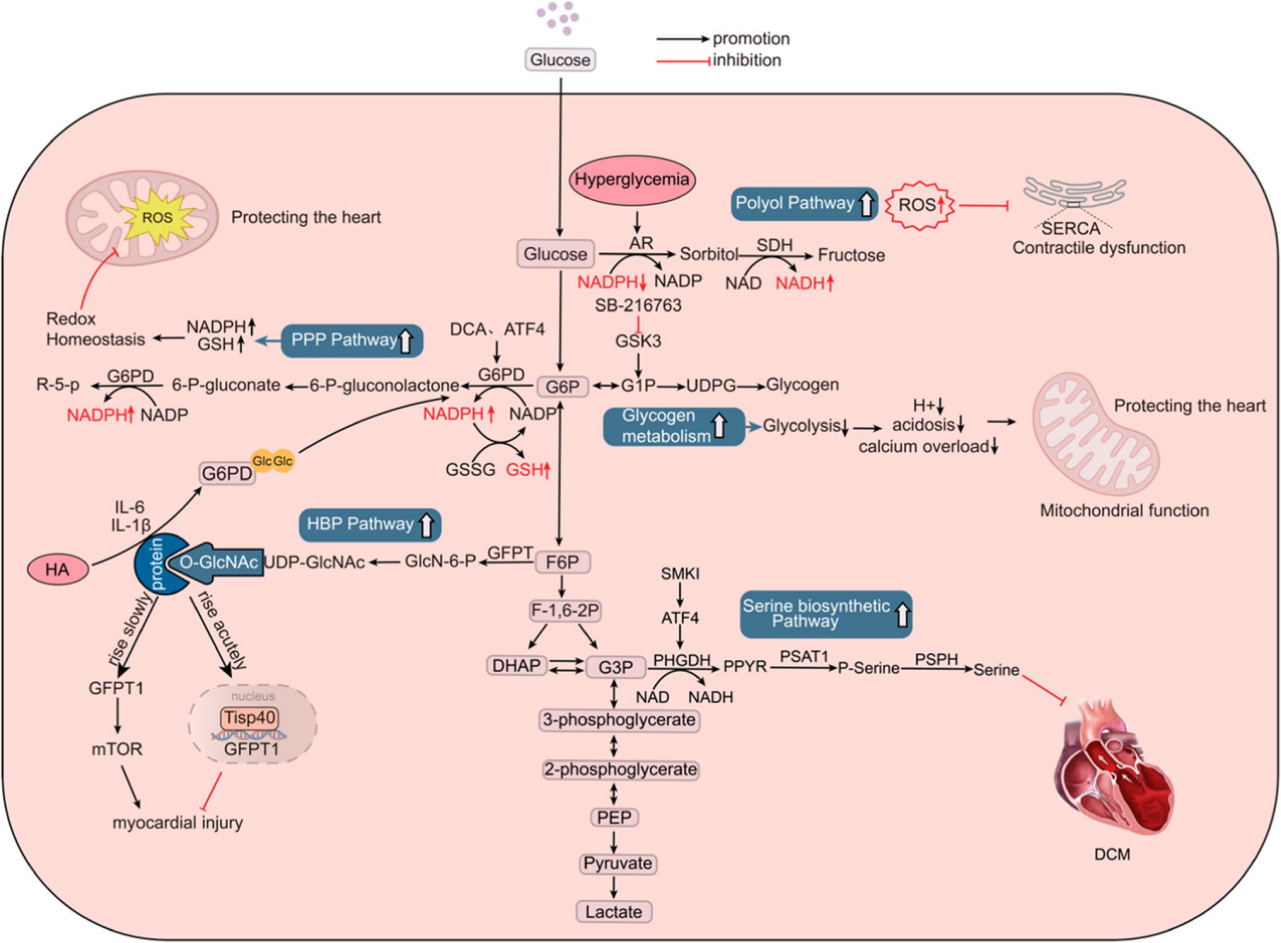
Under hyperglycemic conditions, AR is activated and glucose metabolism is diverted to the Polyol bypass pathway. This leads to a rise in NADPH, a fall in NADH, and an accumulation of ROS, which may further lead to inactivation of SERCA in the sarcoplasm and impaired Ca^2 +^ reuptake, which is associated with myocardial dysfunction.Activation of the PPP bypass pathway and the single-carbon pathway of metabolism increases the concentrations of NADPH and GSH, which maintain intracellular redox homeostasis and protect the heart.Acute activation of the HBP pathway has a cardioprotective effect, and long-term chronic activation of the HBP pathway has a damaging effect on the heart; by inhibiting GSK-1, the HBP pathway is activated, and by inhibiting GSK-1, the HBP pathway is activated. activation of the HBP pathway has a cardioprotective effect, and long-term chronic activation of the HBP pathway has a damaging effect on the heart. Inhibition of G-6-P partitioning by GSK-3 into the glycogen synthesis pathway reduces H production, intracellular acidosis, and calcium overload. Improves mitochondrial function and protects the heart. Serine biosynthesis pathway is associated with the development of DCM. Specific activation of ATF4 using SMKI is able to activate the serine biosynthesis pathway through the activation of PHGDH and attenuate contractile dysfunction in DCM

The serine biosynthesis pathway is an auxiliary branch of the glycolytic pathway that allows for the de novo synthesis of serine using the glycolytic intermediate glyceraldehyde 3-phosphate (G3P) and its eventual conversion to glycine, which provides the carbon unit for single-carbon metabolism [198]. The process involves three enzymes, phosphoglycerate dehydrogenase (PHGDH), phosphoserine transaminase (PSAT1), and phosphoserine phosphorylase (PSPH). Serine is an important nonessential amino acid involved in a variety of physiological processes and pathways. For example, serine is a precursor to glycine and cysteine, and glycine is in turn a biosynthetic precursor to porphyrins. Serine is also involved in purine synthesis, sphingolipid, and phospholipid composition, and is essential for the biosynthesis of macromolecules required for cell proliferation [12, 199]. As a result, the serine biosynthesis pathway has received much attention in the field of cancer research. However, how this pathway functions in cardiovascular disease have not been addressed. Recently, the serine biosynthetic pathway is associated with the onset and progression of hereditary dilated cardiomyopathy [198]. This study found that activation of the ATF4-dependent serine biosynthesis pathway and TRIB4 kinase signaling using a specific combination of small molecule kinase inhibitors (SMKIs) was able to attenuate the dilated cardiomyopathy phenotype in iPSC-CMs by establishing a screening model for dilated cardiomyopathy iPSC-CMs, whereas inhibition of the serine biosynthesis biosynthetic pathway or PHGDH exacerbated contractile dysfunction in dilated cardiomyopathy iPSC-CMs. suggesting that the serine biosynthesis pathway may have a cardioprotective role in dilated cardiomyopathy, but its specific link to dilated cardiomyopathy pathogenesis requires further investigation [198]. In addition, Laura Padrón-Barthe et al. found that CnAβ1 was able to induce ATP synthesis and antioxidant metabolite production through activation of the sericinic acid pathway, resulting in a reduction of GSH production after pressure overload, with beneficial effects on reducing myocardial hypertrophy and improving cardiac function [200]. Overall, activation of the serine biosynthesis pathway appears to be a favorable process for both cardiac physiology and pathophysiology and may serve as an important therapeutic target for cardiovascular disease in the future (Fig. 3).

## Summary and outlook

Cardiovascular disease (CVD) has a high prevalence worldwide and is the leading cause of death in China. With the prevalence of CVD, there is an urgent need to develop unconventional therapeutic tools to continuously improve the level of diagnosis and treatment of CVD. Over the past decades, it has been gradually discovered that glycolytic metabolism plays an indispensable role in several common CVD types (e.g., myocardial infarction, heart failure), and therefore it is crucial to explore the mechanisms of action and therapeutic targets between glycolysis-related enzymes and CVD. Although some of the mechanisms, including how glycolysis-related enzymes protect cardiac structure and function by regulating apoptosis in cardiomyocytes and inducing inducible mitochondrial autophagy, have been reported, the specific functions related to their multiple biological processes remain poorly defined. In this review, we explored the relationship between glycolysis-related enzymes and CVD as much as possible. Among the ten enzymes related to glycolysis, HK is involved in myocardial ischemia–reperfusion and heart failure, PGI is involved in heart failure, PFK is involved in diastolic heart failure, diabetic cardiomyopathy, and coronary artery disease, ALDOA is involved in heart failure, myocardial infarction, arrhythmia, hypertrophic cardiomyopathy, and congenital heart disease and can be used as a serum marker for cardiogenic shock, PGAM is involved in heart failure, ischemia–reperfusion injury, and myocardial infarction, ENO is involved in heart failure, myocardial infarction, diabetic cardiomyopathy and Dox-induced myocardial injury, PKM is involved in myocardial infarction, heart failure, cardiomyopathy and atherosclerosis, and LDH is involved in post-infarction cardiac repair, heart failure and aortic dissection. It is uncertain whether 3-phosphoglyceraldehyde dehydrogenase and phosphoglycerate kinase are involved in CVD. The auxiliary pathways of glycolysis polyol pathway, pentose phosphate pathway, single-carbon metabolism, hexosamine biosynthesis pathway, glycogen metabolism, and serine biosynthesis pathway also play important roles in CVD.

Mechanisms that have been demonstrated in studies of glycolysis-related enzymes include that binding of HK2 to VDAC on the outer mitochondrial membrane inhibits the opening of mPTP and reduces cell death, and that mTORC1-mediated modulation of mitochondrial autophagy promotes mitochondrial homeostasis and reduces the extent of myocardial injury during ischemia–reperfusion. Inhibition of the RIP3-PGAM5-Drp1-mitochondrial pathway was able to achieve myocardial protection by inhibiting necrotic apoptosis. Inhibition of transcriptional activation of ENO1 was able to reduce glycolysis and prevent myocardial fibrosis after MI, among others. It is important to note that most of the signaling pathways and mechanisms identified in these studies were performed in mouse and cellular models, and it is uncertain whether they are equally applicable to human patient tissues. Similarly, activators and inhibitors of the relevant targets have not been tested in clinical trials, and more work is needed to apply basic research findings to clinical settings.
